# Effect of residual volume after surgery of the discoid lateral meniscus on tibiofemoral joint biomechanics: a finite element analysis

**DOI:** 10.1186/s13018-023-04522-w

**Published:** 2024-01-06

**Authors:** Xin Shen, Meifeng Lu, Muzi Liu, Ronghui Xie, Shiguo Gong, Chunjing Yang, Guicai Sun

**Affiliations:** 1 Department of Sports Medicine, Orthopaedic Hospital, The First Affiliated Hospital, Jiangxi Medical College, Nanchang University, No. 17, Yongwai Zhengjie, Donghu District, Nanchang City, Jiangxi Province China; 2 Department of Osteoarthrosis, The First People’s Hospital of Jiujiang, Jiujiang City, Jiangxi Province China; 3 Department of Medical Imaging, The First People’s Hospital of Jiujiang, Jiujiang City, Jiangxi Province China

**Keywords:** Discoid lateral meniscus, Finite element simulation, Residual volume

## Abstract

**Background:**

The purpose of this study was to investigate the influence of different residual meniscus volume on the biomechanics of tibiofemoral joint after discoid lateral meniscus (DLM) surgery by finite element analysis.

**Methods:**

A knee joint model was established based on CT and MRI imaging data. The DLM model was divided into five regions according to conventional meniscectomy, with volumes of 15%, 15%, 15%, 15%, 15%, and 40% for each region. Additionally, the DLM model was divided into anterior and posterior parts to obtain ten regions. The DLM was resected according to the design scheme, and together with the intact discoid meniscus, a total of 15 models were obtained. Finite element analysis was conducted to assess shear and pressure trends on the knee joint.

**Results:**

The study observed significant changes in peak shear stress and compressive stress in the lateral meniscus and lateral femur cartilage. As the meniscus volume decreased, there was an increase in these stresses. Specifically, when the meniscus volume reduced to 40%, there was a sharp increase in shear stress (302%) and compressive stress (152%) on the meniscus, as well as shear stress (195%) and compressive stress (157%) on the lateral femur cartilage. Furthermore, the model grouping results showed that preserving a higher frontal volume in the meniscus model provided better biomechanical advantages.

**Conclusion:**

The use of finite element analysis has demonstrated that preserving more than 55% of the meniscus volume is necessary to prevent a significant increase in joint stress, which can potentially lead to joint degeneration. Additionally, it is crucial to preserve the front volume of the DLM in order to achieve improved knee biomechanical outcomes.

## Introduction

The discoid lateral meniscus is a congenital variation of the knee joint, affecting its morphology and structure and potentially leading to meniscal instability [1]. Due to its unique morphology and anatomical features, the discoid lateral meniscus is more prone to injury, resulting in symptoms such as pain, popping, or limited extension [2, 3]. In cases of symptomatic discoid lateral meniscus, surgical intervention is typically needed to alleviate knee joint symptoms and preserve meniscal function to the greatest extent possible. As the importance of the meniscus has gained increasing recognition, preserving the sufficient width and height of the discoid meniscus, restoring its normal shape, and ensuring the stability of the remaining meniscus are considered essential for maintaining its physiological function and protecting the knee joint [1, 4–6].

Regarding meniscus preservation, many studies utilize meniscus width as a criterion, and some scholars advocate retaining a lateral meniscus width of 6–8 mm as a preferable choice [4, 7–9]. According to Liu Wenlong et al., the width of the discoid lateral meniscus should be preserved at 8–10 mm through finite element study. Jamison G Gamble et al. found that the remaining width of the discoid meniscus should be at least 10 mm for an 8-year-old child and at least 15 mm for an adolescent, based on the normal width of the meniscus [11]. Some researchers also believe that the width of the intermediate part of the medial meniscus should be regarded as a reference point for the margin of the remaining meniscus [1, 12]. None of the aforementioned studies mentioned the use of residual volume to evaluate the postoperative meniscus. In our study, we focused on the remaining meniscus as the subject of research, taking into consideration its width, thickness, and specific morphology. Additionally, we conducted an in-depth analysis of the biomechanical changes in the tibiofemoral joint following discoid lateral meniscus surgery. It is anticipated that this study will serve as a foundation and provide insights for the surgical treatment of discoid lateral meniscus.

With advancements in computer and related software technology, the finite element method has rapidly developed and plays a crucial role in the field of orthopedic biomechanics. It has become a valuable tool for understanding the biomechanical properties of the human body, allowing for the creation of models that closely resemble normal specimens in terms of structure and yielding significant findings in meniscus research [10, 13–19}. In this study, we will establish all components of the knee joint model (including bone, meniscus, cartilage and ligament) by computer, design meniscus models with different residual volumes, and then add corresponding loads and boundary constraints to analyze the biomechanical changes in the knee joint.

## Materials and methods

Principles of the Finite Element Analysis: The finite element analysis is the use of mathematical approximation to the real physical system (geometry and load conditions) simulation, also using simple and interacting elements, namely, the unit, it is possible to use a finite number of unknowns to approximate the infinite number of unknowns of the real system. The basic idea is to discretize the elastic region, express the displacement of any node in the element by function, establish the element equation, then integrate the element into the node and add the external load force, at the same time, introduce the displacement boundary conditions for solving, obtain the node displacement, and obtain the element strain and stress according to the elastic mechanics formula. In this study, we use this simulation method to calculate the shear stress and compressive stress we need.

### Data acquisition

An adult female volunteer with CDLM, aged 25 years, height 162 cm, and weight 49 kg, was selected for the study. Knee diseases besides CDLM were ruled out based on history inquiry, physical examination, CT, and MRI of the knee joint. The volunteers agreed and signed an informed consent form for the study. We scanned the patient's knee joint with 256-row Siemens CT, and the scan section thickness was 0.625 mm. Additionally, Siemens 3.0 T-MRI was used to scan the patient's knee joint, and the scan section thickness was 1 mm. DICOM data were obtained from both the CT and MRI scans.

### Reconstruction of the 3D knee joint model

**Fig. 1:**
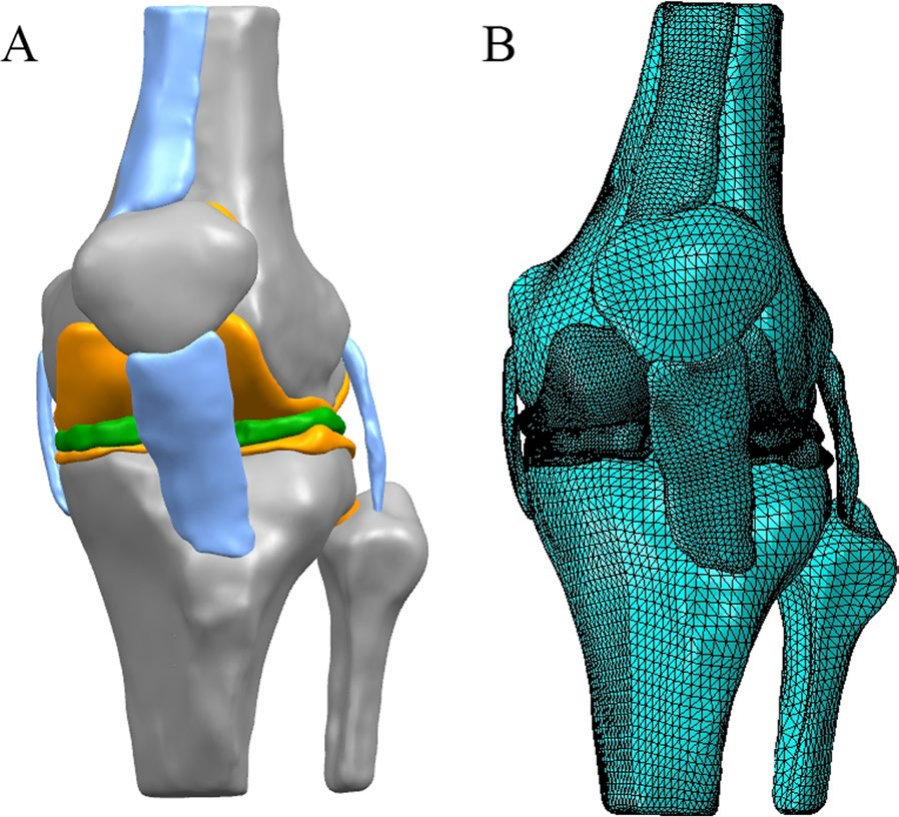
The view of 3D models used in the FE simulation. **A** A general view of knee joint. **B** 3D model for mechanical analysis in ANASYS software

CT and MRI data were imported into Mimicis21.0, appropriate gray values were selected in CT to distinguish bones from surrounding soft tissues, and bone models including femur, patella, tibia and fibula were extracted and generated. For the MRI scans, a manual segmentation process was employed to extract the contours of the meniscus, cartilage, anterior and posterior cruciate ligaments, medial and lateral collateral ligaments, patellar ligament, and quadriceps tendon. This segmentation process was conducted in pairs and supervised by experienced orthopedic surgeons and radiologists, ensuring an accuracy of 0.1 mm to minimize any model discrepancies. Additionally, measurements were taken on the meniscus model to ensure consistency with the MRI scans. The data were saved in STL format and imported into Geomagic Wrap2017 software for reverse engineering reconstruction technology processing, and a complete 3D knee joint model was established, as shown in Fig. 1A. The three-dimensional knee joint model was imported into ANASYS 18.0 software. The femur, tibia and fibula were reconstructed using shell units with a mesh size of 2.0 mm, the articular cartilage and meniscus were reconstructed using tetrahedral cells (C3D4) of 1.0 mm [20, 21], and the model shown in Fig. 1B was obtained.

**Fig. 2:**
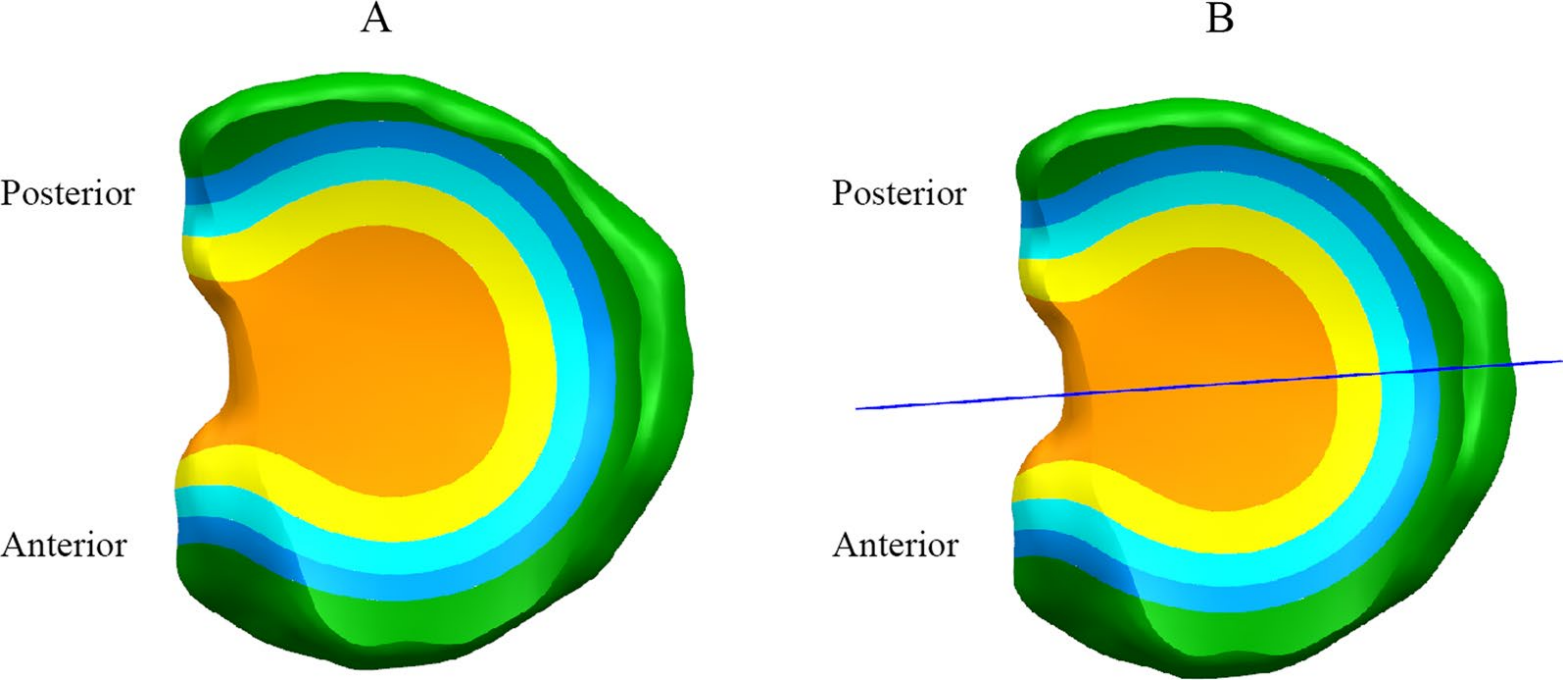
Scheme for division of the meniscus model. **A** Division of the discoid meniscus by volume ratio: Orange (15%), yellow (15%), light blue (15%), blue (15%), and green (40%). **B** Division line dividing the discoid meniscus into anterior and posterior sections

**Fig. 3:**
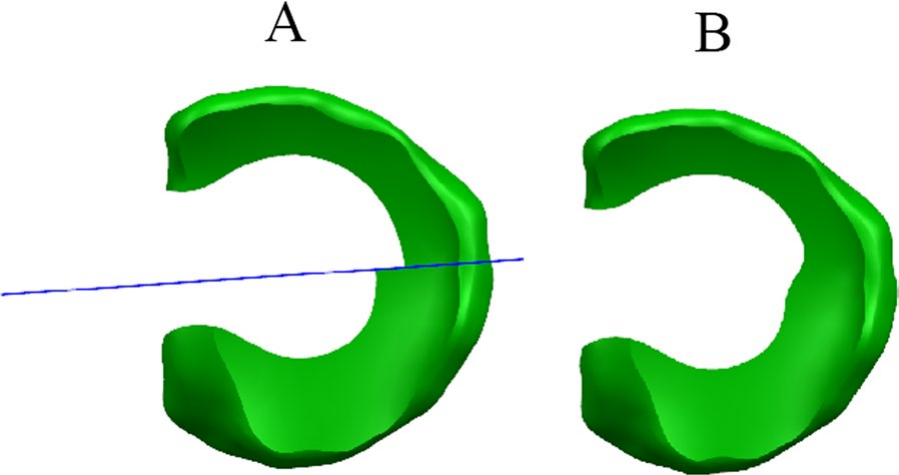
Image processing of an anterior–posterior unequally resected meniscus model. **A** Model of anterior and posterior unequally resected meniscus. **B** Model after smoothing

**Fig. 4:**
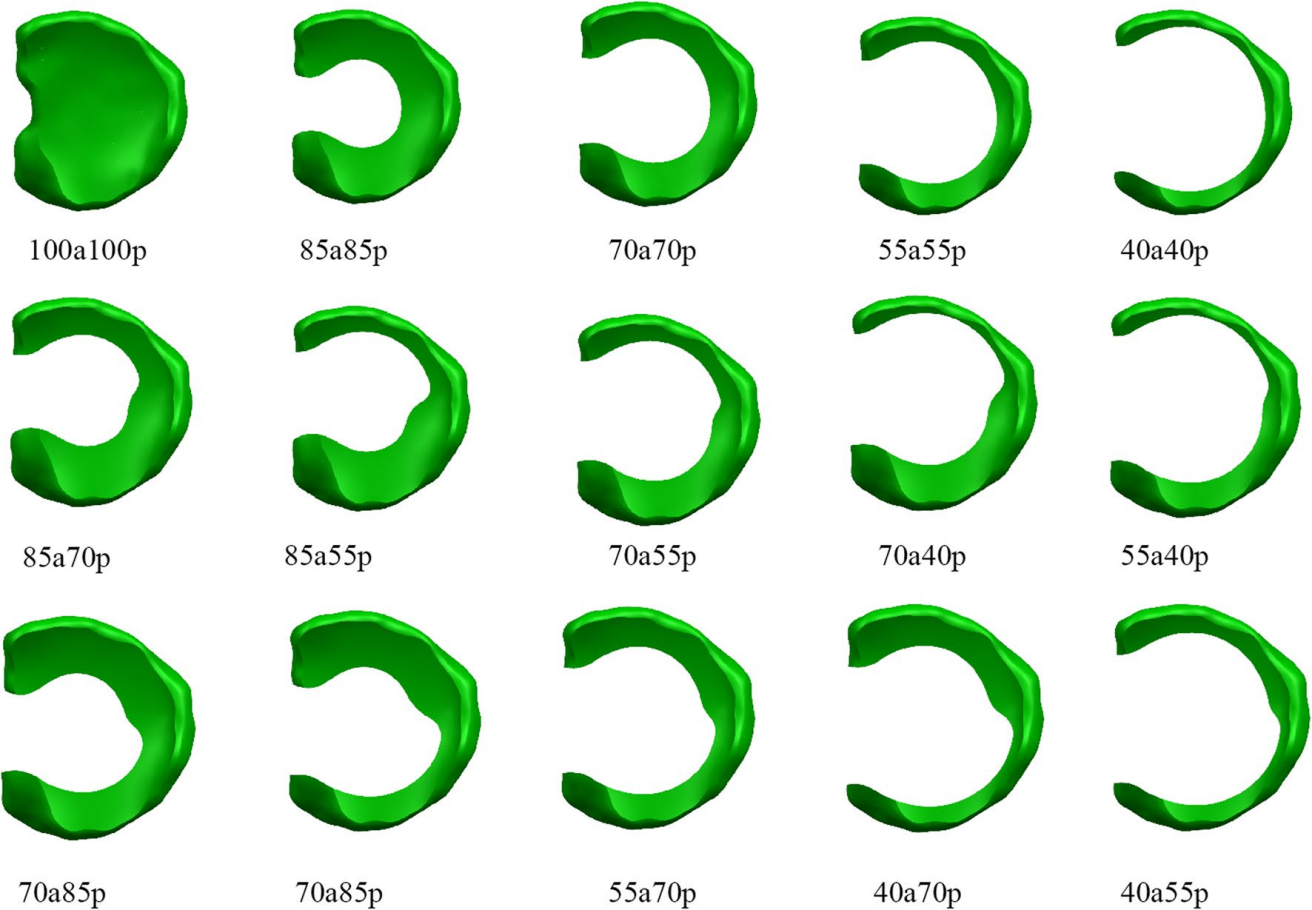
Various models of lateral meniscus according to designs

The meniscus model was processed by calculating the total volume of the meniscus and dividing it into different regions based on the volume ratio. The meniscus was also divided into anterior and posterior parts (Fig. 2). By resecting different regions of the meniscus, various models were obtained. The resulting meniscus models were named based on the ratio of the remaining volume of the anterior to posterior meniscus. For example, if the remaining volume of the anterior was 85% and the remaining volume of the posterior was 70%, the model would be named 85a70p. This naming convention was used for all the models generated. The models included variations such as 100% remaining volume in both the anterior and posterior (100a100p), 85% remaining volume in both the anterior and posterior (85a85p), and 70% remaining volume in both the anterior and posterior (70a70p). A total of 15 models were generated. The models with differences in the anterior and posterior portions had smooth transitions in the union of the two parts (Fig. 3). All the meniscus models are shown in Fig. 4.

### Materials and properties

We refer to the parameters in other finite element studies of the knee joint to assign values to the various parts of the knee joint model. We set bone as a rigid material [17] and articular cartilage as an isotropic linear elastic material [18]. The materials of the anterior and posterior cruciate ligaments are set as Neo-Hookean hyperelastic materials, and other ligament materials are set as isotropic linear elastic materials[16, 18]. The meniscus provided resistance against shear forces within the joint cavity and prevented outward expansion. Consequently, the meniscus exhibits less horizontal deformation compared to longitudinal deformation when subjected to joint loading [19, 20]. Therefore, we set the meniscus as a cross sectional isotropic material and set different elastic moduli in the circumferential, axial and radial directions [10, 20]. All material data can be found in Table 1.

**Table 1:** Material properties of each component of the knee joint model

	Material properties	Modulus of elasticity (MPa)	Poisson’s ratio	Shear modulus C1	Non-shear shrinkage parameter D1
Bone	Rigidity	–	–	–	–
Articular cartilage	Isotropic linear elasticity	15	0.3	–	–
Anterior Cruciate Ligament	Superelastic	–	–	5.08	0.00683
Posterior cruciate ligament	Superelastic	–	–	6.06	0.0041
Medial and lateral collateral ligaments	Isotropic linear elasticity	60	0.3	–	–
Patellar ligament/quadriceps tendon	Isotropic linear elasticity	60	0.3	–	–
Meniscus	Cross sectional isotropic	Circumferential 140 Radial 20 Axial 20	In-plane 0.2 Out-of-plane 0.3	–	–

### Boundary conditions and loads

We implemented fixation for the tibia–fibula, limiting the flexion, anteroposterior translation, and axial rotation of the femur. In addition, we allowed unrestricted varus and valgus movements, as well as medial and lateral translation and axial translation of the femur. To secure the ligaments and meniscus, their ends and roots were fixed in contact with the bone structure. We restrained the contact surfaces between bone and cartilage, as well as between bone and ligament. For the contact between cartilage and cartilage and between cartilage and meniscus, we established limited sliding, frictionless, and nonpermeable hard contact. Furthermore, we applied a vertical compressive load of 1150 N on the femur [16, 19, 20].

## Results

**Fig. 5:**
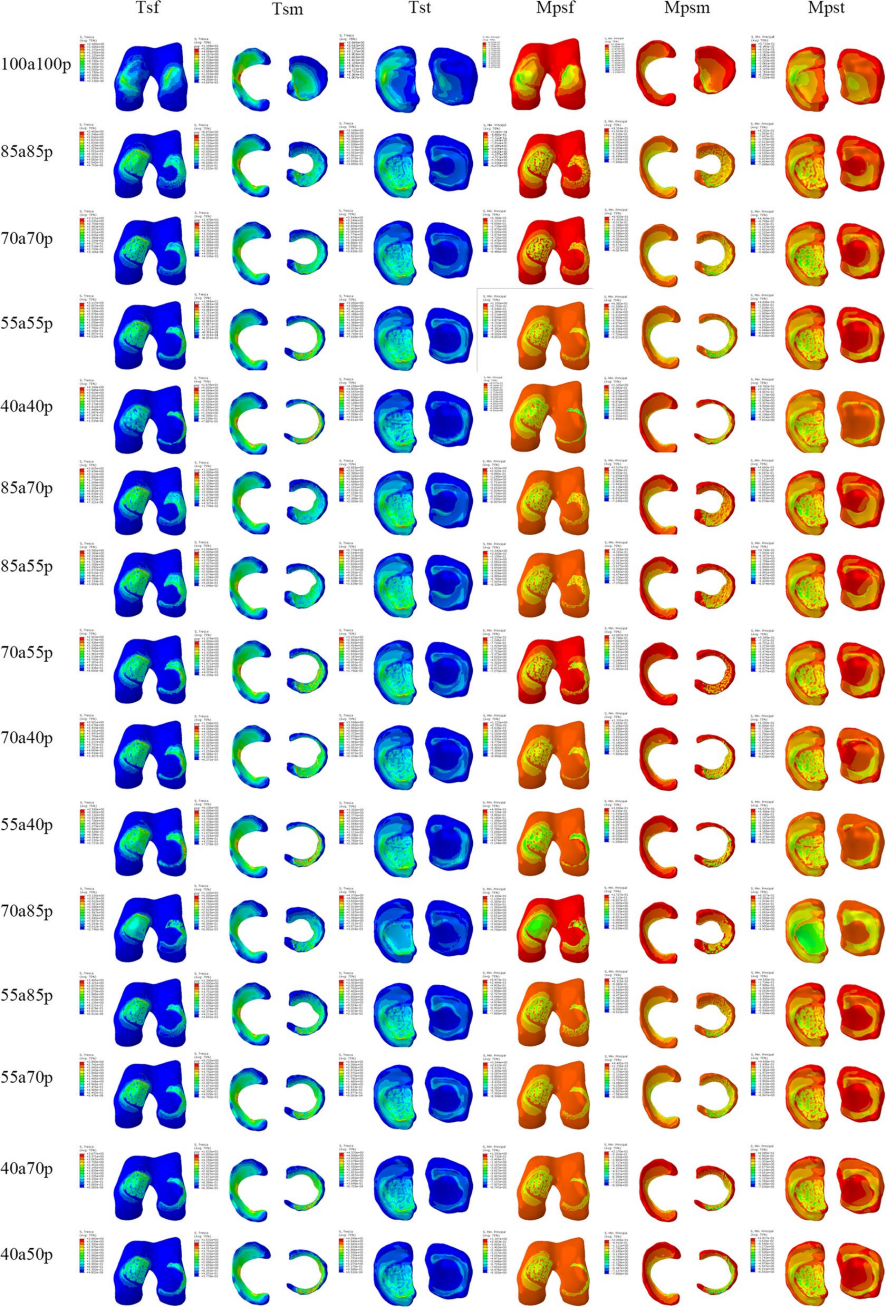
The results of shear stress and compressive stress on the femoral cartilage, meniscus and tibial cartilage under static stance simulation. Tsf: tresca stress (shear stress) of the femoral cartilage; Tsm: tresca stress(shear stress) of the meniscus; Tst: tresca stress (shear stress) of the tibial cartilage; Mpsf: minimum principal stress of the femoral cartilage; Mpsm: minimum principal stress of the meniscus; Mpst: minimum principal stress of the tibial cartilage

In this study, we obtained 15 lateral meniscus models based on our design. These models allowed us to directly observe the morphology of the lateral meniscus remnants. Each model was fitted into the knee joint model and analyzed using finite element analysis (FEA). The main focus of our analysis was on the Tresca stress (shear stress) and maximum compressive stress (minimum principal stress). The obtained stress nephograms are presented in Fig. 5. We analyzed the results according to three different series.

### Variation with meniscus residual volume ratios

**Fig. 6:**
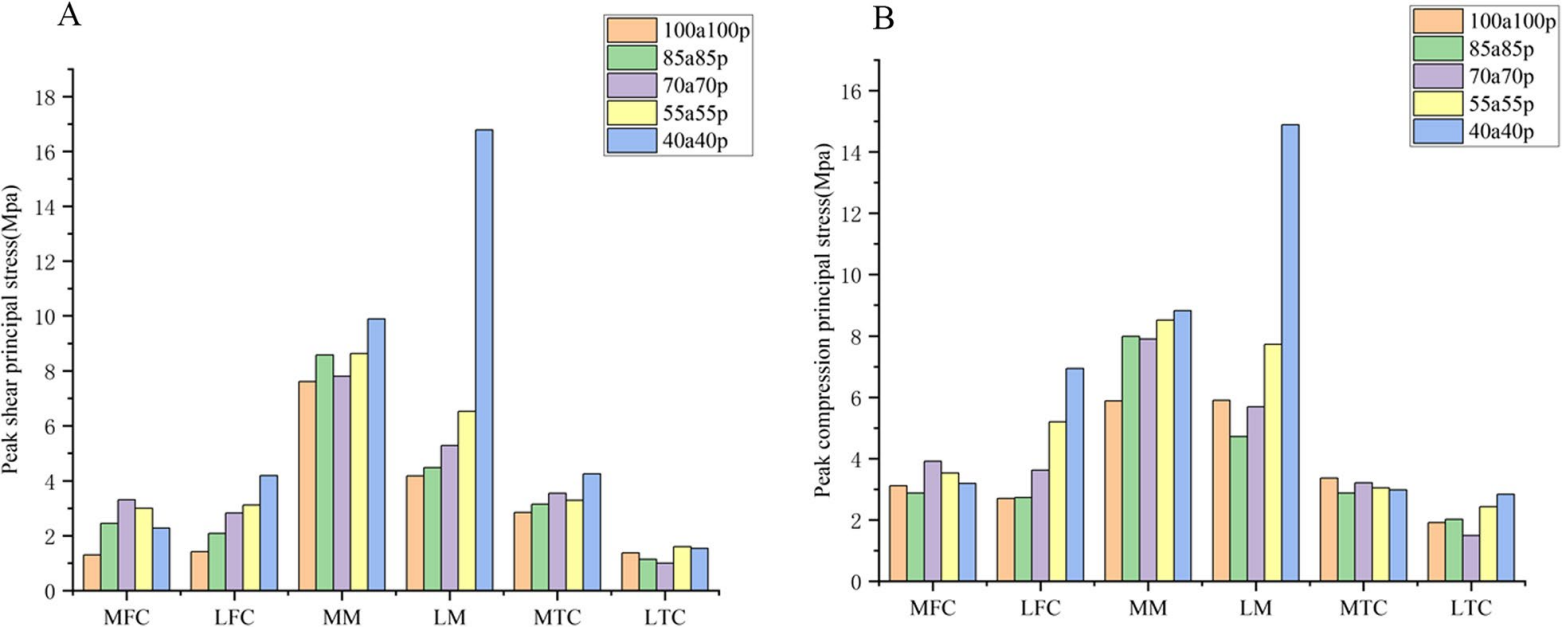
**A** Peak shear principal stress applied on the knee joint with different meniscus models. **B** Peak compression principal stress (min principal stress) on the knee joint with different meniscus models MFC: medial femoral cartilage; LFC: lateral femoral cartilage; MM: medial meniscus; LM: lateral meniscus; MTC: medial tibial cartilage; LTC: lateral tibial cartilage

We selected knee models with different meniscus residual volume ratios (100%, 85%, 70%, 55%, and 40%) to observe the variation in shear stresses and minimum principal stresses (Fig. 6). The lateral meniscus (LM) showed the greatest changes in shear stress and minimum principal stress compared to the medial femoral condyle (MFC), lateral femoral condyle (LFC), medial meniscus (MM), medial tibial cartilage (MTC), and lateral tibial cartilage (LTC). As the volume of the lateral meniscus decreased from 100 to 40%, the peak shear stress on the lateral meniscus increased by 302%, from 4.175 MPa to 16.78 MPa. This significant increase in shear stress was mainly observed in the 40a40p model. Furthermore, the minimum peak principal stress on the lateral meniscus was significantly elevated to 14.88 MPa in the 40a40p model, which was 152% higher than that in the intact discoid meniscus. The shear stress and minimum principal stress on the cartilage of the lateral femoral condyle also showed a gradual increase, with increases of 195% and 157% compared to the intact discoid meniscus, respectively. The shear force and minimum principal stress on the medial meniscus showed a small increase; while, the shear force on the medial femoral condylar cartilage showed a tendency to increase and then decrease. No clear pattern was observed in the changes in shear force and minimum principal stress in the medial and lateral tibial cartilage.

### Comparison within groups with the same remaining volume of meniscus and anterior–posterior partial differential resection

**Fig. 7:**
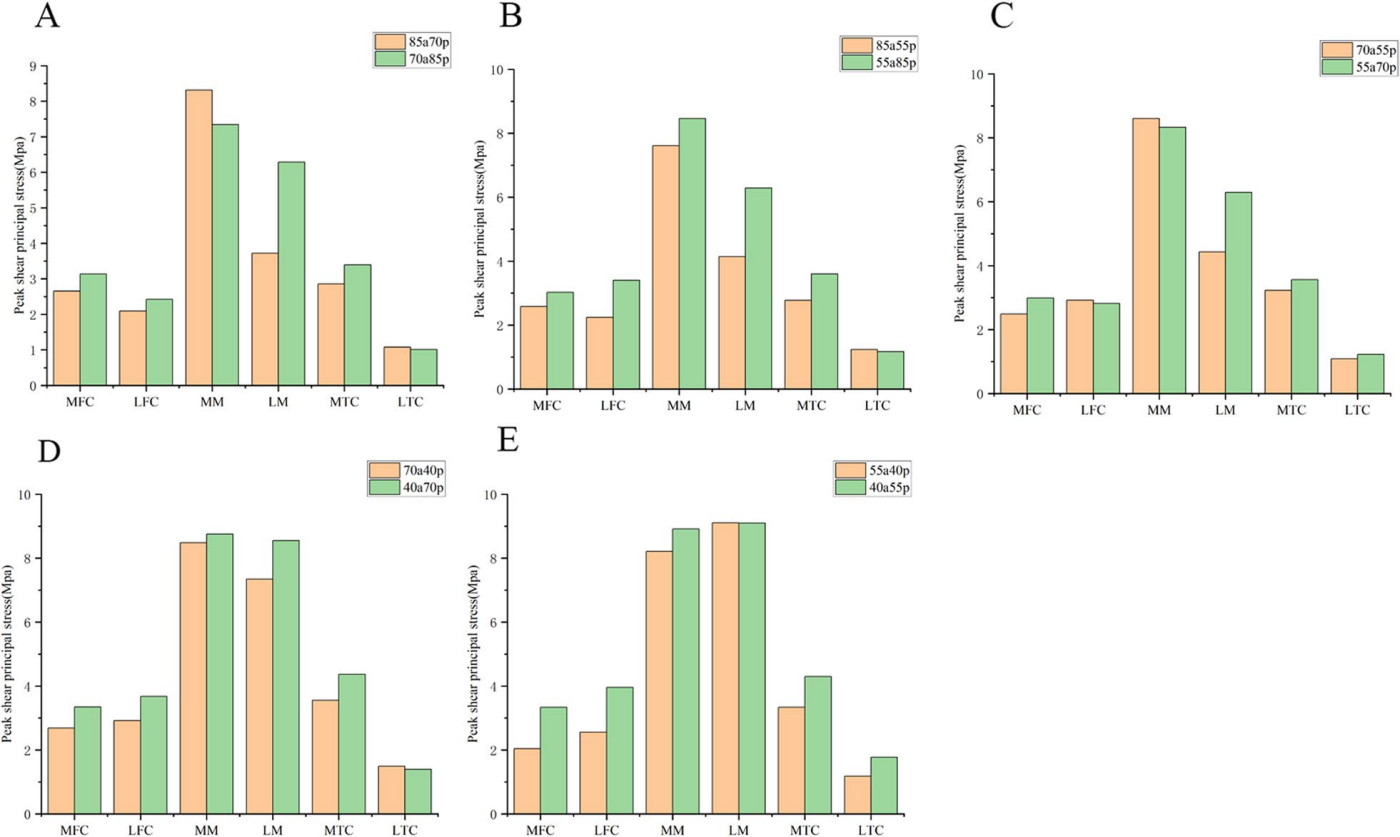
Peak shear principal stress applied on the knee joint in 5 groups. **A** 85a70p and 70a85p. **B** 85a55p and 55a85p. **C** 70a55p and 55a70p. **D** 70a40p and 40a70p. **E** 55a40p and 40a55p MFC: medial femoral cartilage; LFC: lateral femoral cartilage; MM: medial meniscus; LM: lateral meniscus; MTC: medial tibial cartilage; LTC: lateral tibial cartilage

**Fig. 8:**
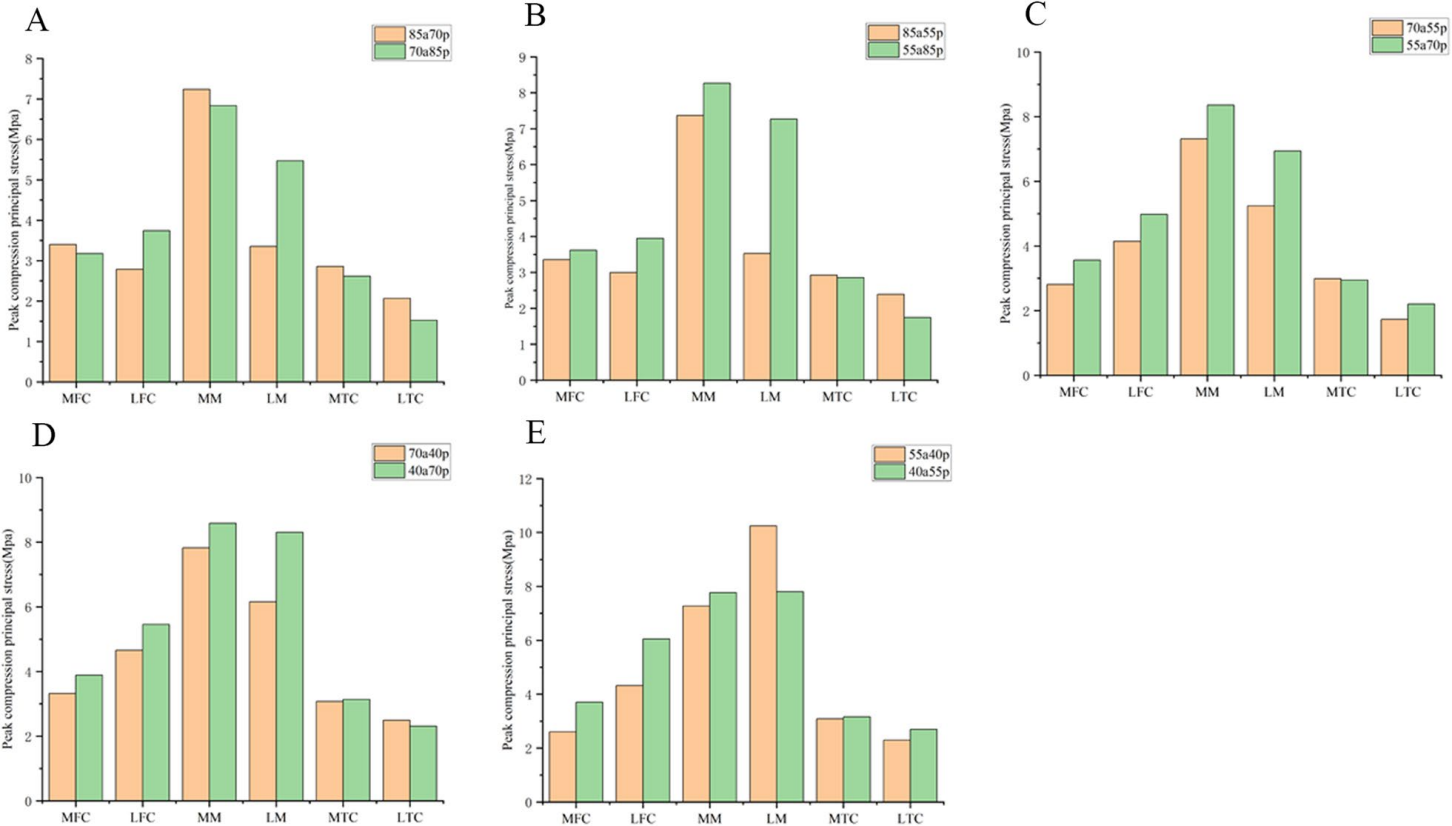
Peak compression principal stress applied on the knee joint in 5 groups. **A** 85a70p and 70a85p. **B** 85a55p and 55a85p. **C** 70a55p and 55a70p. **D** 70a40p and 40a70p. **E** 55a40p and 40a55p MFC: medial femoral cartilage; LFC: lateral femoral cartilage; MM: medial meniscus; LM: lateral meniscus; MTC: medial tibial cartilage; LTC: lateral tibial cartilage

We selected knee models within each group that had the same remaining volume of meniscus and anterior–posterior partial differential resection. We compared the shear stresses and minimum principal stresses within each group (Figs. 7 and 8). The largest differences in within-group comparisons were observed in the shear and minimum principal stress forces on the lateral meniscus. This was particularly evident in group A, group B, group C, and group D. The models with more anterior meniscus showed smaller values of shear and minimum principal stresses. In group E, there was no difference in the shear stress force data on the lateral meniscus, but the minimum principal stresses on the 55a40p model were greater than those on the 40a55p model. This was a significant difference from the results of the other four groups. The overall trend in shear stress and minimum principal stresses on the cartilage of the medial and lateral femoral condyles was that the modeled values for the meniscus with more preservation of the anterior portion were generally smaller. However, there was no clear pattern in the values of shear stress and minimum principal stress in the medial meniscus and medial and lateral tibial cartilage.

### Biomechanical changes with the volume of one part decreased while the other remained unchanged

**Fig. 9:**
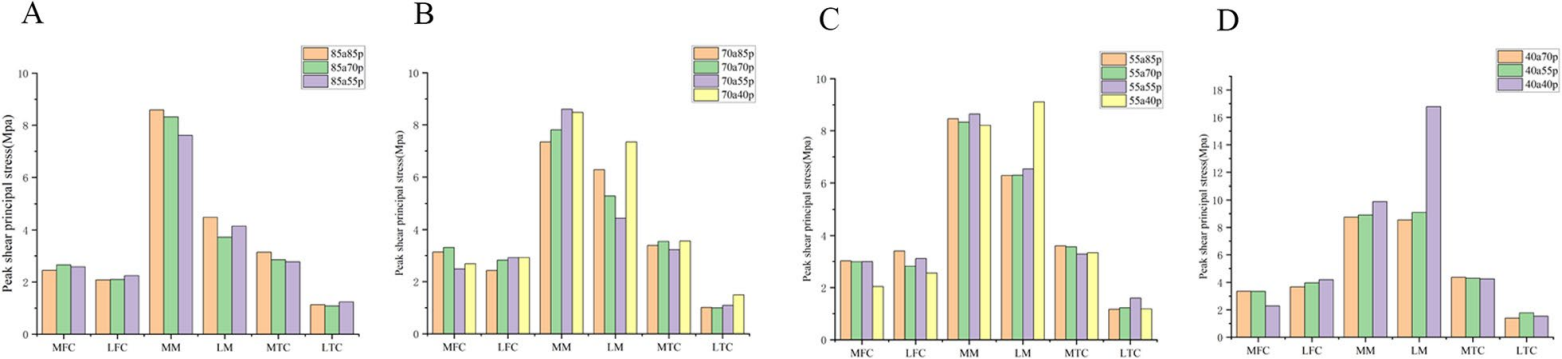
Peak shear principal stress applied on the knee joint in 4 groups. **A** 85a85p,85a70p,85a55p. **B** 70a85p,70a70p,70a55p,70a40p. **C** 55a85p,55a70p,55a55p,55a40p. **D** 40a70p,40a55p,40a40p MFC: medial femoral cartilage; LFC: lateral femoral cartilage; MM: medial meniscus; LM: lateral meniscus; MTC: medial tibial cartilage; LTC: lateral tibial cartilage

**Fig. 10:**
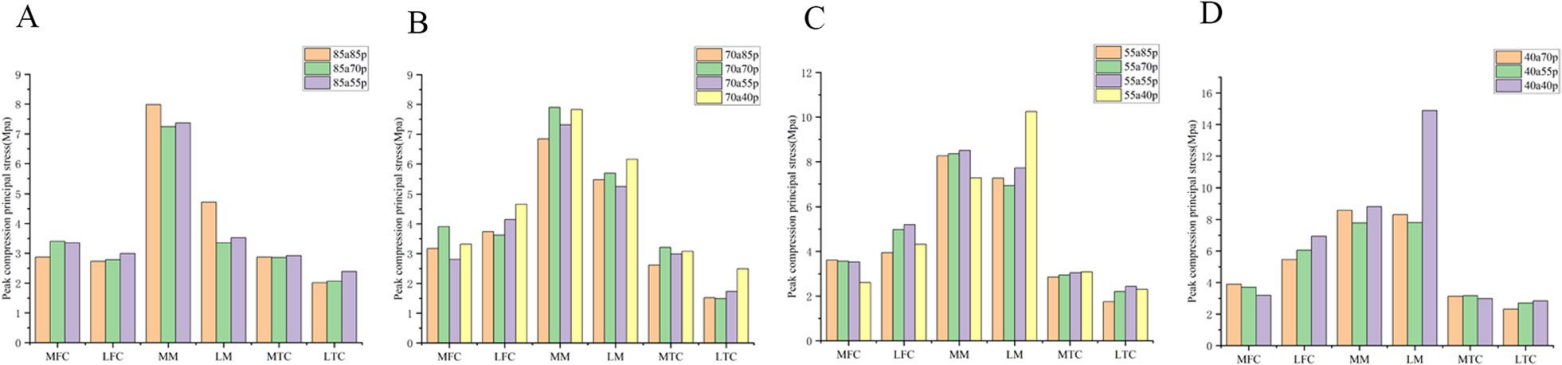
Peak compression principal stress applied on the knee joint in 4 groups. **A** 85a85p,85a70p,85a55p. **B** 70a85p,70a70p,70a55p,70a40p. **C** 55a85p,55a70p,55a55p,55a40p. **D** 40a70p,40a55p,40a40p MFC: medial femoral cartilage; LFC: lateral femoral cartilage; MM: medial meniscus; LM: lateral meniscus; MTC: medial tibial cartilage; LTC: lateral tibial cartilage

**Fig. 11:**
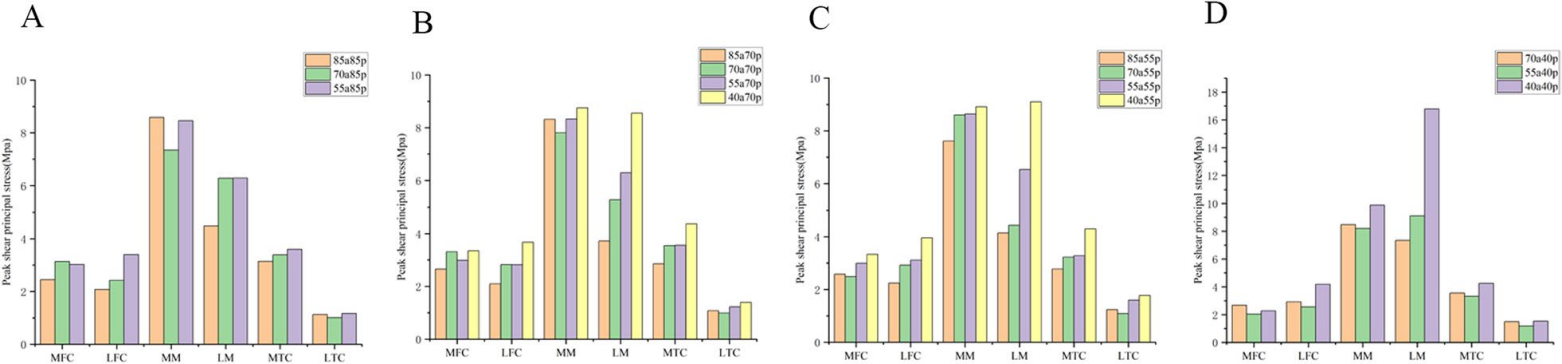
Peak shear principal stress applied on the knee joint in 4 groups. **A** 85a85p,85a70p,85a55p. **B** 70a85p,70a70p,70a55p,70a40p. **C** 55a85p,55a70p,55a55p,55a40p, **D** 40a70p,40a55p,40a40pMFC:medial femoral cartilage; LFC: lateral femoral cartilage; MM: medial meniscus; LM: lateral meniscus; MTC: medial tibial cartilage; LTC: lateral tibial cartilage

**Fig. 12:**
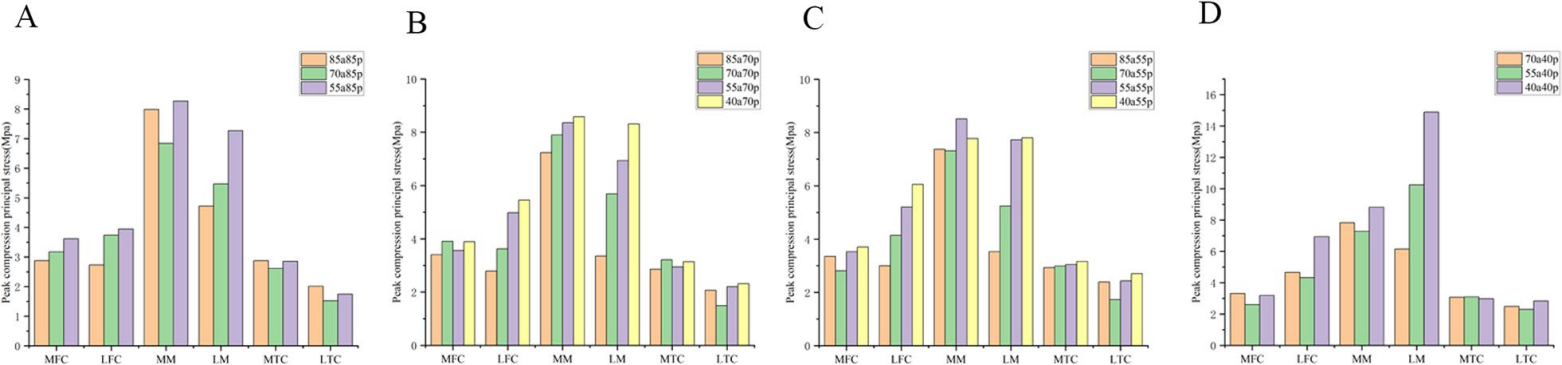
Peak compression principal stress applied on the knee joint in 4 groups. **A **85a85p,85a70p,85a55p. **B** 70a85p,70a70p,70a55p,70a40p. **C** 55a85p,55a70p,55a55p,55a40p. **D** 40a70p,40a55p,40a40p MFC: medial femoral cartilage; LFC: lateral femoral cartilage; MM: medial meniscus; LM: lateral meniscus; MTC: medial tibial cartilage; LTC: lateral tibial cartilage

To obtain different groups of models, we kept the volume of either the anterior or posterior part of the meniscus unchanged while sequentially reducing the volume of the other part. We observed biomechanical changes as the volume of the anterior or posterior part gradually decreased (Figs. 9, 10, 11, and 12). When the volume of the anterior part of the meniscus remained unchanged and the posterior part was reduced, we observed a stepwise upward trend in shear stress and minimum principal stress on the lateral femoral condyle (LFC) and lateral meniscus (LM), as shown in Figs. 11 and 12. However, this trend was not observed for shear stress and minimum principal stress on the lateral femoral cartilage (LFC) and lateral meniscus (LM), as shown in Figs. 9 and 10. In Figs. 9B–D and 10B–D, the shear stresses and minimum principal stresses on the LM showed a clear tendency to increase for the 70a40p, 55a40p, and 40a40p models. The other values within the ABCD group were similar and did not show a clear pattern overall. This may be because when the volume of the posterior meniscus reaches 40%, it significantly affects the peak values of shear stress and minimum principal stress on the entire meniscus. We observed that when the volume of the posterior meniscus was constant, the larger the volume of the anterior portion was, the smaller the shear stresses and minimum principal stresses on the LFC and LM. Similarly, when the volume of the anterior portion was constant, the volume of the posterior portion decreased from 85 to 55%, and the overall trend of the minimum principal stresses on the femoral cartilage, tibial cartilage, and meniscus was not obvious. However, when the volume of the posterior portion reached 40%, the values of shear stresses and minimum principal stresses on the LM were significantly higher.

## Discussion

Through comparison with previous literature on finite element studies of the knee joint [14, 15], it was found that the results of shear stress and compressive stress of femoral cartilage, meniscus and tibial cartilage were similar, thus verifying the validity of this model (Table 2).

**Table 2:** Model validation in different Studies(Mpa)

Studies	SSFC	SSM	SSTC	CSFC	CSM	CSTC
Our model	1.42	7.61	2.85	3.11	5.90	3.36
Zhi xu et al	1.72	11.45	3.56	6.51	6.61	8.45
Zhang K et al	2.00	6.72	2.40	4.25	9.15	6.81

SSFC peak shear principal stress on femoral cartilages; SSM peak shear principal stress on meniscus; SSTC peak shear principal stress on tibial cartilage; CSFC peak compression principal stress on femoral cartilages; CSM peak compression principal stress on meniscus; CSTC peak compression principal stress on tibial cartilages

In this study, we utilized computer software to calculate the volume of the meniscus and simulated 15 meniscus models. We then calculated the shear force and minimum principal stress values of the meniscus and articular cartilage for each model in the extended standing position using finite element analysis. Our results indicated that the discoid lateral meniscus exhibited the best biomechanical state when intact, which aligns with Takuji Yokoe's findings [13]. Additionally, as the volume of the lateral discoid meniscus gradually decreased isometrically, the changes in shear force and pressure on the lateral meniscus and the lateral femoral condyle cartilage were more pronounced. Shear stresses and pressures increased gradually with meniscus reduction, with a significant increase occurring when the meniscus volume reached 40%. Therefore, it is crucial to preserve a larger volume of meniscus during partial lateral disc meniscectomy. We suggest that preserving 85% of the volume ensures that biomechanical parameters are similar to those of an intact meniscus and maintains a shape similar to that of a normal meniscus. Furthermore, it is important to preserve as much meniscus volume as possible within the range of 85% to 55%, as shear stresses and pressures increase significantly at 40% of the remaining volume.

We performed volume segmentation at ratios of 15%, 15%, 15%, 15%, 15%, and 40%. This decision was based on the observation that the volume of tissue surrounding the meniscus was proportionally large. Additionally, at a volume of less than 40%, the width of the meniscus became so small, approximately 4 mm from the edge, that further segmentation was deemed impractical.

One aspect of this study focused on the biomechanical effects of unequal anterior and posterior meniscus volumes. We designed 5 pairs of models with equal total volume but differing anterior and posterior volumes. The maximum difference in volume between the anterior and posterior portions was 30%, reflecting the clinical practice of preserving a similar proportion of the anterior and posterior meniscus. Comparing the total volume with the anterior–posterior volume, we found that models with a larger anterior volume exhibited better biomechanical effects. This result may be attributed to the fact that, in the standing position, the peak contact of the lateral meniscus occurs in the anterior horn area [22–24]. Hence, preserving the anterior meniscus plays a more critical role. Comparing the biomechanical changes when the anterior meniscus volume remained constant but the posterior meniscus volume gradually decreased with the reverse scenario, we observed that, with a gradual reduction in the anterior meniscus, the shear and pressure on the lateral femoral condyle and lateral meniscus increased in a stepwise manner when the posterior meniscus volume remained unchanged. Conversely, we did not observe a similar pattern when the anterior meniscus volume gradually decreased with a constant posterior meniscus volume. This finding suggests that changes in the anterior meniscus volume are closely related to the effects of shear force and pressure on the lateral femoral condyle and lateral meniscus, further emphasizing the importance of preserving the anterior meniscus.

Due to the thicker discoid meniscus, discoid meniscectomy creates a significant gap in the outer joint space, altering the knee joint's biomechanical stress distribution. Several follow-up studies have reported complications such as secondary knee degeneration, posterolateral instability of the knee joint, and knee valgus [25–27]resulting from discoid meniscectomy. Therefore, preserving an adequate width of the remaining peripheral edge of the discoid lateral meniscus is crucial. Despite numerous studies focusing on meniscus width, there is currently no clear consensus on the degree of preservation of the peripheral edge and the anatomical references that should be considered [2]. Unlike previous studies that solely examined meniscus width, our study focused on meniscus volume. We employed 3D reconstruction technology to comprehensively evaluate the status of the remaining meniscus, providing guidance for surgical treatment and prognostic assessment of the discoid lateral meniscus. In clinical practice, meniscus preservation may not always achieve an equal proportion in every part due to various factors, including meniscus quality, tear type, and tear location. In such cases, the volume of the remaining meniscus may offer a more graphic representation than the meniscus width. According to the methodology of this study, the status of the remaining meniscus on the three-dimensional reconstructed meniscus can be clearly understood. Based on the relevant findings of this study, it is suggested that clinicians can utilize computerized three-dimensional reconstruction techniques in the surgical treatment of discoid meniscus with a remaining meniscus volume of less than 55% to avoid abnormal joint stresses, particularly in the lateral compartment, which can lead to joint degeneration. This approach may help reduce the risk of osteoarthritis. However, if the remaining meniscus volume is less than 40% due to various factors, a poor prognosis is expected for the patient, and surgical treatments such as lateral meniscus replacement can be considered at a later stage. During the operation, it is important to pay close attention to the preservation of the anterior horn, as it can better protect the stress distribution of the knee joint post-surgery. In cases where excessive damage to the anterior horn requires greater meniscus resection, stress concentration in the anterior horn is likely to occur, and close observation of the patient's knee joint degeneration is necessary. Therefore, as computerized quantitative calculation abilities and three-dimensional reconstruction technology continue to improve, assessing the volume of the remaining meniscus as a postoperative evaluation method for discoid meniscectomy becomes more advantageous. Although this assessment method currently requires significant time in clinical practice and is less convenient than using meniscus width, it offers valuable insights.

However, this study has several limitations. First, computer simulation methods have inherent drawbacks, including potential accuracy loss when extracting geometry from medical images and inevitable shape distortions during processing. Although we processed the data model to reflect the characteristic curvature of the bone, meniscus, and cartilage, the geometry error remains indeterminate due to multiple improvements in the initial geometry. Therefore, this will impact our final results to some extent. The advancement of computer simulation technology can further minimize this error, and we can also utilize additional model data to support error reduction. Second, we only performed simulations on a single knee joint with different discoid meniscus models to evaluate biomechanics under static vertical loading. However, the stresses on the discoid meniscus dynamically change during knee joint activity and are more complex. Our findings are not confined to the standing position, which could potentially impact the experimental outcomes. Therefore, it is essential to create multiple disc-shaped meniscus models with varying shapes and simulate the force distribution under different angles of knee flexion as well as internal and external inversion. This will enhance the robustness of our conclusions. Third, when creating discoid meniscus models with unequal anterior and posterior volumes, we had to smooth the junction between the anterior and posterior parts, which may introduce errors in the anterior and posterior volume ratios and subsequently affect the experimental results. We can measure the residual meniscus volume through specific clinical cases and then classify it for related finite element analysis. This approach can provide a closer integration with clinical practice and help to reduce errors. Finally, the research results presented here were solely obtained through finite element analysis without the support of mechanical experiments using human specimens. This limitation may affect the accuracy of the experiments. In future studies, it would be beneficial to include human specimens that meet our experimental conditions to improve the investigation of human mechanical behavior. Additionally, designing clinical retrospective studies can further enhance the credibility of the experimental findings.

Overall, despite these limitations, this study provides valuable insights into the preservation of meniscus volume during partial meniscectomy, particularly in cases of discoid lateral meniscus. The findings contribute to the understanding of the biomechanical effects and offer guidance for surgical treatment and prognostic assessment. Further research and validation are necessary to enhance the clinical application of these findings.

## Conclusion

In conclusion, this study emphasizes the importance of preserving meniscus volume during partial meniscectomy to maintain optimal biomechanical parameters in the knee joint. The study suggests preserving more than 55% of the meniscus volume to minimize stress on the tibiofemoral joint. The study recommends preserving the volume of the anterior portion of the discoid meniscus, as it is particularly important. Based on our findings, we can propose a multi-faceted approach in the future to assess the postoperative condition of the discoid lateral meniscus. This approach would provide more precise information, enabling clinicians to devise appropriate treatment plans and determine prognosis more effectively.

## Declaration

## Data Availability

The datasets used and/or analyzed during the current study are available from the corresponding author upon reasonable request.
